# A novel Lugol’s iodine staining technique to visualize the upper margin of the surgical anal canal intraoperatively for Hirschsprung disease: a case series

**DOI:** 10.1186/s12893-020-00986-3

**Published:** 2020-12-04

**Authors:** Kazuki Yokota, Hizuru Amano, Toyoki Kudo, Takeshi Yamamura, Yujiro Tanaka, Takahisa Tainaka, Chiyoe Shirota, Wataru Sumida, Satoshi Makita, Aitaro Takimoto, Masanao Nakamura, Mitsuhiro Fujishiro, Akinari Hinoki, Hiroo Uchida

**Affiliations:** 1 Department of Pediatric Surgery, Nagoya University Graduate School of Medicine, 65 Tsurumai-cho, Showa-ku, Nagoya, 466-8550 Japan; 2 Digestive Disease Center, Showa University Northern Yokohama Hospital, 35-1 Chigasakichuo, Tsuzuki-ku, Yokohama, 224-8503 Japan; 3 Department of Gastroenterology and Hepatology, Nagoya University Graduate School of Medicine, 65 Tsurumai-cho, Showa-ku, Nagoya, 466-8550 Japan

**Keywords:** Hirschsprung disease, Aganglionosis, Lugol’s iodine staining, Surgical anal canal, Transanal pull-through

## Abstract

**Background:**

In cases of Hirschsprung disease, complete and reproducible resection of the aganglionic bowel is ideal to achieve good postoperative bowel function. Reliable identification of the upper margin of the surgical anal canal, which is the squamous-columnar junction, is necessary during transanal pull-through. Here, we describe a novel staining technique using Lugol’s iodine stain to visualize the upper margin of the surgical anal canal.

**Methods:**

Lugol’s iodine staining was performed in five patients with Hirschsprung disease treated using a single-stage laparoscopic transanal pull-through modified Swenson procedure. In two of these patients, endocytoscopic observation with ultra-high magnification was performed using methylene blue and crystal violet to mark the border of the squamous epithelium at 1 week before surgery. The alignment between the incisional line, which was revealed using Lugol’s iodine staining and endocytoscopic marking, was evaluated. Complications, including postoperative bowel dysfunction, were evaluated.

**Results:**

In all cases, Lugol’s iodine staining produced a well-demarcated line. The endocytoscopic marking of the upper margin of the surgical anal canal was aligned with the line revealed by Lugol’s iodine staining. There were no complications associated with the transanal pull-through procedure, including postoperative bowel dysfunction.

**Conclusions:**

Lugol’s iodine staining could be a safe and practical method to visualize the upper margin of the surgical anal canal intraoperatively. This finding may be useful for surgeons to make a consistent removal of the aganglionic bowel during surgery for Hirschsprung disease.

## Background

**Fig. 1:**
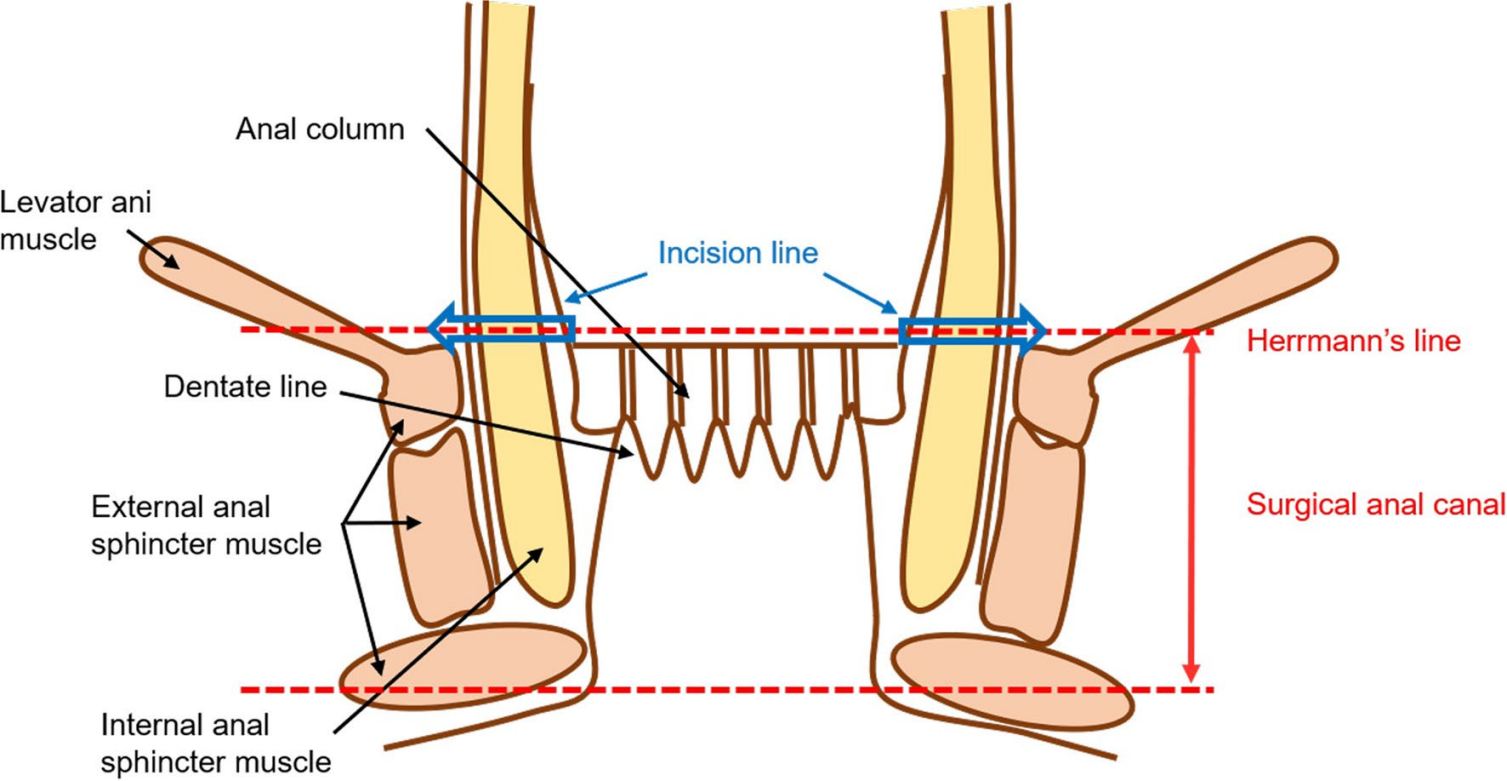
Anatomy of the anal canal

Resection of the aganglionic bowel is one of the definitive treatments for patients with Hirschsprung disease (HD), followed by reconstruction using a transanal pull-through of the ganglionic bowel. Complete removal of the aganglionic bowel without injuring the entire surgical anal canal, including the innervated anal transition zone, is crucial to ensure postoperative bowel function. [1–4] Generally, in our institution, transanal circumferential full-thickness dissection is performed from the upper margin of the surgical anal canal during a single-stage laparoscopic transanal pull-through modified Swenson procedure (Fig. 1). [5] However, without establishing a standard method for identifying the upper margin of the surgical anal canal, this boundary is detected intraoperatively through visual inspection or palpation, regardless of the surgeon’s experience. [6]

Histologically, the upper margin of the surgical anal canal defines the boundary between the stratified squamous epithelium and simple columnar epithelium, [7] corresponding to the superior margin of the levator ani muscle. [5, 8] To identify this line, we focused on Lugol’s iodine staining, which is a popular technique to stain the normal non-keratinized squamous epithelium used in the diagnosis of esophageal cancer. Oono et al. reported that Lugol’s iodine staining was useful for identifying a squamous cell carcinoma of the anal canal in situ demarcated as an unstained area with Lugol chromoendoscopy. [9] However, there are no reports of the use of Lugol’s iodine staining in surgery for HD. Το the best of our knowledge, this is the first case series report, in which Lugol’s iodine staining was used to detect the upper margin of the surgical anal canal during surgery for HD.

## Methods

This study aimed to describe a novel staining technique using Lugol’s iodine stain to visualize the upper margin of the surgical anal canal. Between February and October 2019, five patients with HD (including four and one cases of short- total-segment, respectively) were treated using the single-stage laparoscopic transanal pull-through modified Swenson procedure [5] at Nagoya University Hospital, Nagoya, Japan.

Briefly, after performing a full-thickness bowel biopsy to confirm the correct position of the ganglionic intestine, laparoscopic dissection was extended to the peritoneal reflection of the rectum. The rectum below the peritoneal reflection was circumferentially dissected up to the superior border of the levator ani muscle. After placement of an anal retractor (The Lone Star Retractor System™; Yufu, Tokyo, Japan), transanal circumferential full-thickness dissection was performed from the upper margin of the surgical anal canal, without leaving a muscular cuff (Fig. 1). The ganglionic bowel was pulled through and anastomosed to the anal canal.

In all five patients, Lugol’s iodine staining was performed intraoperatively, before the transanal circumferential full-thickness dissection. Lugol's solution containing iodine 1% (10 mg/mL) and potassium iodide 2% (20 mg/mL) was used. After washing out the rectal mucus with saline, 1 mL of Lugol's solution was sprayed onto the anal canal and the rectum each time for a total of approximately 5–10 mL. When the stain was inadequate, gauze soaked with Lugol's solution was placed on the mucosa for approximately 1 min. In two cases, endocytoscopy with 1% methylene blue and 0.05% crystal violet staining [10] was performed at 1 week before the radical surgery to mark the upper margin of the surgical anal canal. Endocytoscopy is a novel ultra-high magnification technique that allows microscopic observation at the cellular level using intraprocedural stains. In this technique, methylene blue clearly stains the cell nuclei, whereas the crystal violet stains the cytoplasm, allowing the rapid detection of the glandular structure. Therefore, the boundary between the squamous epithelium and columnar epithelium, corresponding to the upper margin of the surgical anal canal, was marked.

At the time of radical surgery performance, we evaluated the alignment between the endocytoscopy marking. The incisional line revealed using Lugol’s iodine staining with direct intraoperative visualization. Complications, including postoperative bowel dysfunction, were evaluated.

## Results

**Fig. 2:**
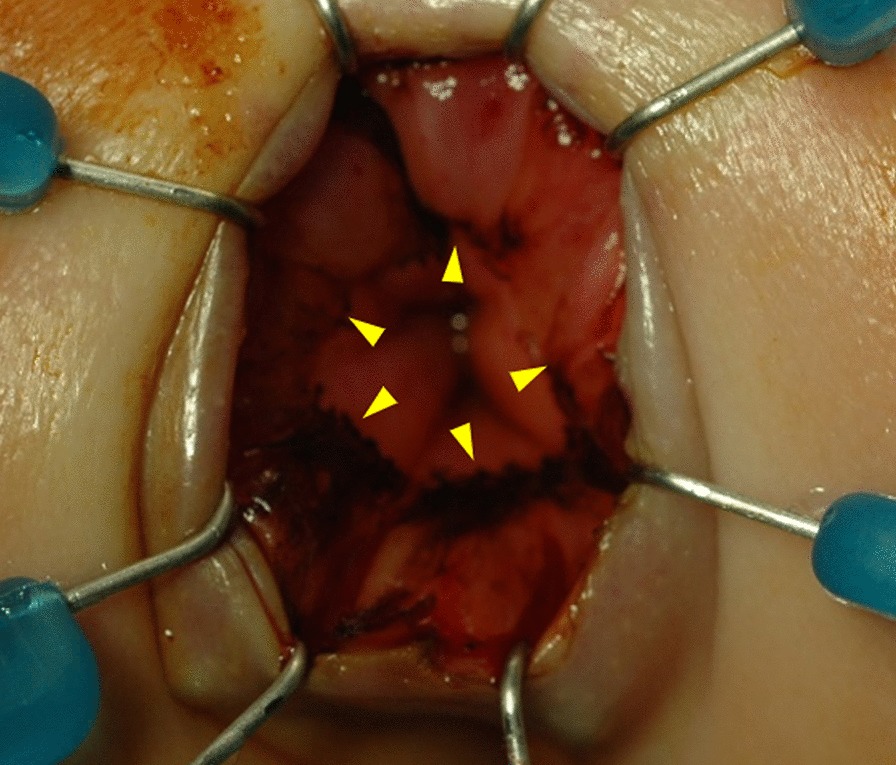
Lugol’s iodine staining, showing a well-demarcated line, indicated by arrowheads

**Fig. 3:**
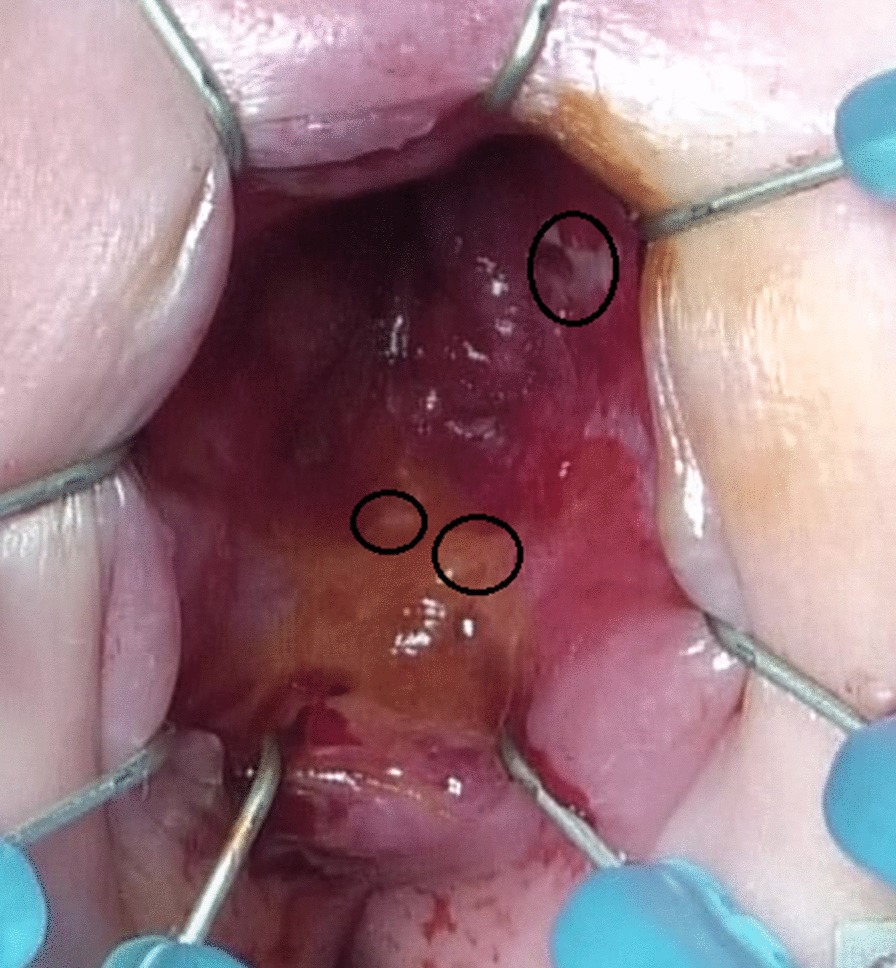
Upper margin of the surgical anal canal. The upper margin of the surgical anal canal marked using endocytoscopy (black ovals) coincided with the line demarcated with Lugol’s iodine staining

**Fig. 4:**
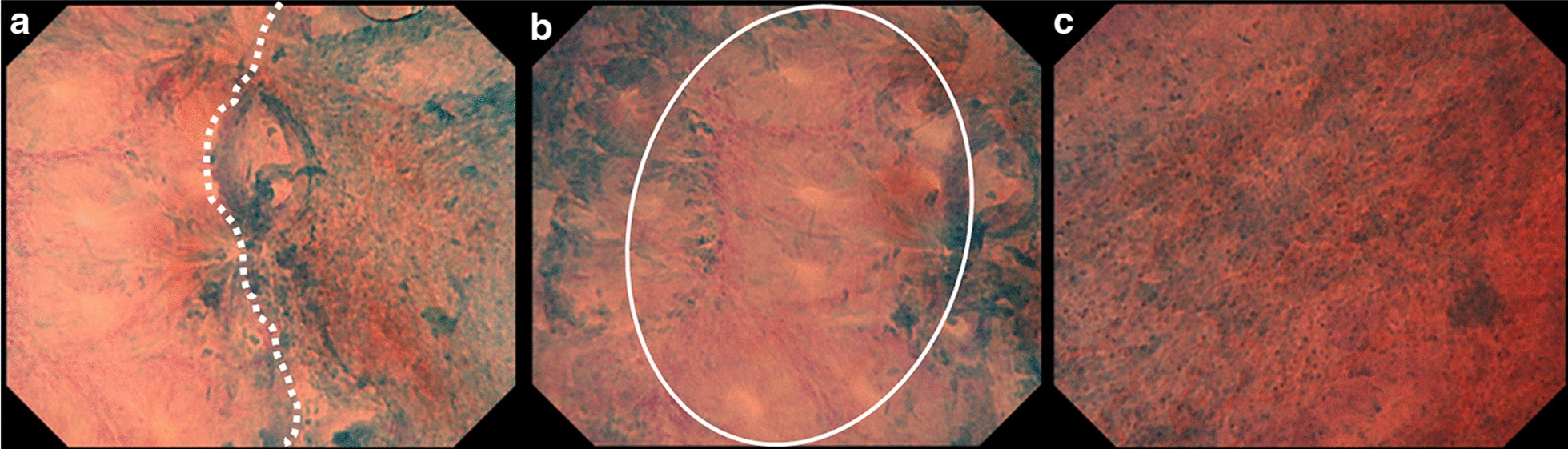
**a** A representative endocytoscopic image, showing the boundary (white dotted line) between the simple columnar epithelium and stratified squamous epithelium. **b** The simple columnar epithelium has a small regular nucleus at the marginal region of the glandular structure (a white oval). **c** The stratified squamous epithelium has a small nucleus with cytoplasm-rich cells, in a regular arrangement

Lugol’s iodine staining revealed a well-demarcated line, indicating the squamous-columnar junction in all patients (Fig. 2). In the two cases where we used endocytoscopy, the squamous-columnar junction marked using endocytoscopy (i.e., the upper margin of the surgical anal canal) coincided with the line demarcated with Lugol’s iodine staining. The latter indicated that Lugol's iodine staining could detect the upper margin of the surgical anal canal accurately at the cellular level (Figs. 3 and 4).

We removed the aganglionic bowel in all five patients after full-thickness circumferential dissection completion. Pathological findings of the rectal stump showed no squamous epithelium in four patients and a slight squamous epithelium in one patient. No intra- or postoperative complications were noted, including postoperative bowel dysfunction (e.g., enterocolitis or constipation).

## Discussion

The upper margin of the surgical anal canal is the circular upper border of the puborectalis (levator ani) muscle (Fig. 1). Full-thickness dissection along line indicates complete resection of the aganglionic bowel. Lugol’s iodine staining, which was previously described, was easily applied and identified the upper margin of the surgical anal canal as the distinct boundary between the squamous epithelium and columnar epithelium in the anorectal region. This was confirmed through endocytoscopy with ultra-high-power magnification at the cellular level. [10] Therefore, we recommend Lugol’s iodine staining as a reliable and easy method to detect the upper margin of the surgical anal canal.

To the best of our knowledge, this is the first report describing a reliable method to visualize the upper margin of the surgical anal canal during surgery. Our method enables precise detection of the starting point for mucosal (Soave procedure) or full-thickness resection (our modified Swenson procedure). Using Lugol’s iodine staining, reconstruction with transanal pull-through of the ganglionic bowel could be performed from a repeatable starting position. Such reproducibility in reconstruction would allow us to compare postoperative bowel function across different operative procedures.

The safety of Lugol’s iodine staining has been previously described in the endoscopic diagnosis for esophageal cancer. Lugol’s staining can be easily performed intraoperatively, with no special technique. Lugol’s staining can be easily introduced within the intraoperative procedures performed in any hospital. This method of visualizing the upper margin of the surgical anal canal is expected to allow surgeons start an incision on the same line during surgery for HD. This reliability in identifying the incisional line would lower the risk for postoperative fecal continence because anal sensation is preserved while the aganglionic bowel, proximal to the upper margin of the surgical anal canal, is excised. In our limited five-case report, there was no occurrence of postoperative bowel dysfunction, including enterocolitis or constipation. Long-term follow-up is required to evaluate more completely the effect of this method on postoperative bowel function.

## Conclusions

Lugol’s iodine staining provides a useful method for detecting the upper margin of the surgical anal canal during surgery for HD, based on the short-term follow-up findings of postoperative bowel function in five patients with HD. Because the number of cases was limited in this study, a thorough evaluation with a larger sample size is needed in the future.

## Data Availability

Not applicable.

## Abbreviation

HD: Hirschsprung disease
